# A Measure for Mothers: Model Predicts Lactational Transfer of PCB-153

**Published:** 2008-12

**Authors:** M. Nathaniel Mead

Breastfed infants sit at the top of the food chain for the simple reason that their nourishment comes from other humans. Through biomagnification, environmental chemicals such as polychlorinated biphenyls (PCBs) are passed up the food chain to the nursling. Although epidemiologic studies have established an association between prenatal PCB exposures and neurodevelopmental and neurobehavioral problems, the potential health risks of xenobiotic exposures via human milk are less clear and remain an area of intense research interest [see “Contaminants in Human Milk: Weighing the Risks against the Benefits of Breastfeeding,” *EHP* 116:A426–A434 (2008)]. Researchers have now developed a physiologically based pharmacokinetic model of PCB-153 in women to predict the transfer of this compound via lactation **[*EHP*** **116:1629–1634; Redding et al.]**.

PCB-153 was selected for study because it is the most prevalent PCB congener in human tissues. To predict the concentration of PCB-153 in human milk, physiological parameters were obtained from a Taiwanese cohort and from reference values in published studies. Partition coefficients were estimated based on chemical structure and the lipid content in various body tissues as reported in the literature: liver, fat, mammary tissue, and the “rest of the body” (an average of brain, skin, and muscle), as well as a mixed blood compartment.

The investigators predicted the acquired body burden of PCB-153 from birth over a 25-year period on the basis of estimates of exposure via diet using data from Japanese population studies. They then compared the model’s predictions with measurements from published studies in multiple countries.

Blood and tissue concentrations for a 25-year-old woman generated by the model were found to fall within ranges reported in the literature, assuming that dietary intake of PCB-153 was the principal source of this chemical in human milk. Additionally, the researchers demonstrated the use of the model for reverse dosimetry, also referred to as “exposure reconstruction,” for possible exposure scenarios in Canadian Inuits, who consume extremely high levels of PCB-153 through their traditional high-fat diet.

This human, population-scale lactational model for PCB-153 is the first to successfully predict a range of results that encompass human biomonitoring data of milk PCB-153 content from all over the world. The primary value of this model will be its ability to describe the distribution, absorption, metabolism, and elimination of PCB-153 in nursing women. The new tool could also be useful for reverse dosimetry modeling to enable retrospective analyses of potential health effects of PCB exposures in breastfed individuals.

